# Supplementary material for: Short-lived metal-centered excited state initiates iron-methionine photodissociation in ferrous cytochrome c
Source: Nat Commun. 2021 Feb 17;12:1086. doi: 10.1038/s41467-021-21423-w (PMC7889893; doi:10.1038/s41467-021-21423-w)
Supplement: Supplementary file 1 — Supplementary Information [file 41467_2021_21423_MOESM1_ESM.pdf]

Short-lived metal-centered excited state initiates iron-methionine photodissociation in ferrous cytochrome *c*

Supplementary Information

Marco E. Reinhard[1,2], Michael W. Mara[3,§], Thomas Kroll[2], Hyeongtaek Lim[3], Ryan G. Hadt[3,^], Roberto Alonso-Mori[4], Matthieu Chollet[4], James M. Glownia[4], Silke Nelson[4], Dimosthenis Sokaras[2], Kristjan Kunnus[1], Tim Brandt van Driel[4], Robert W. Hartsock[1], Kasper S. Kjaer[1], Clemens Weninger[4], Elisa Biasin[1], Leland B. Gee[3], Keith O. Hodgson[2,3], Britt Hedman[2], Uwe Bergmann[1], Edward I. Solomon[2,3*], Kelly J. Gaffney[1,2*]

[1]PULSE Institute, SLAC National Accelerator Laboratory, Stanford University, Stanford, CA 94305, USA

[2]Stanford Synchrotron Radiation Lightsource, SLAC National Accelerator Laboratory, Stanford University, Menlo Park, CA 94025, USA

[3]Department of Chemistry, Stanford University, Stanford, CA 94305, USA

[4]Linac Coherent Light Source, SLAC National Accelerator Laboratory, Stanford University, Menlo Park, CA 94025, USA

Present addresses:

[§]Department of Chemistry, Northwestern University, Evanston IL 60208, United States

[^]Division of Chemistry and Chemical Engineering, Arthur Amos Noyes Laboratory of Chemical Physics, California Institute of Technology, Pasadena, California 91125, United States

*Corresponding authors:

E-mail: edward.solomon@stanford.edu, kgaffney@slac.stanford.edu

Supplementary Note 1: Fe Kβ XES data analysis

To identify any potential intermediates between the photoexcited $^1\pi{-}\pi^*$ state and the ^{5}MC state associated with the five-coordinated heme site, we have applied singular value decomposition (SVD) of the Kβ XES data. If SVD of the Kβ XES difference map is performed in the -0.15 – 0.85 ps range (Supplementary Fig. 1), subsequent reconstruction of the dataset requires two components, but the second component only contributes at delays <0.4 ps, thus supporting the necessity for an intermediate between the $^1\pi{-}\pi^*$ and ^{5}MC states. Performing an SVD in the larger range of -0.65 – 20 ps with coarser time step (Supplementary Fig. 2), a single component previously identified as the quintet minus singlet difference spectrum[1] fully reconstructs the data.

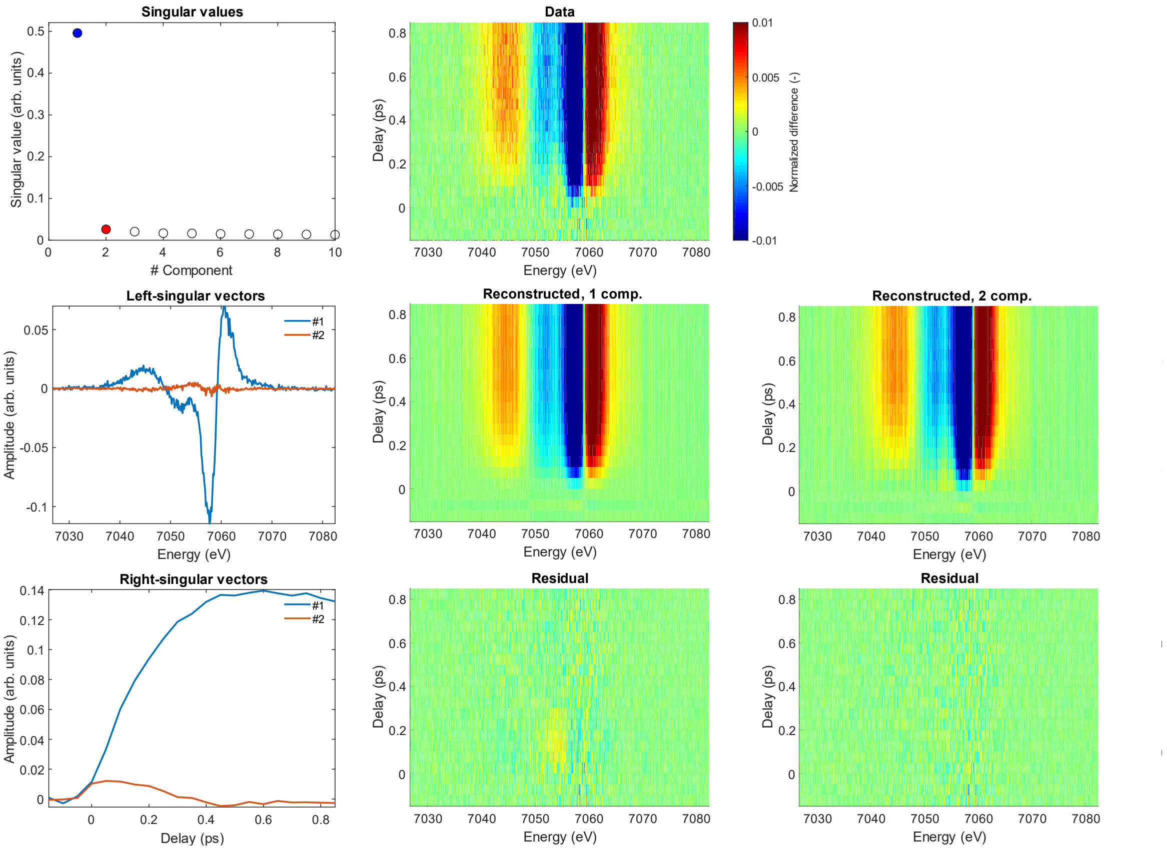

Supplementary Fig. 1. Singular value decomposition of the Kβ XES difference map in the -0.15 – 0.85 ps range. The left column shows the first ten singular values and the first two left-/right-singular vectors. The middle column shows the measured dataset, the reconstructed dataset using only the first left-/right-singular vectors and the residual. The right column shows the reconstructed dataset and residual when two left-/right-singular vectors are used.

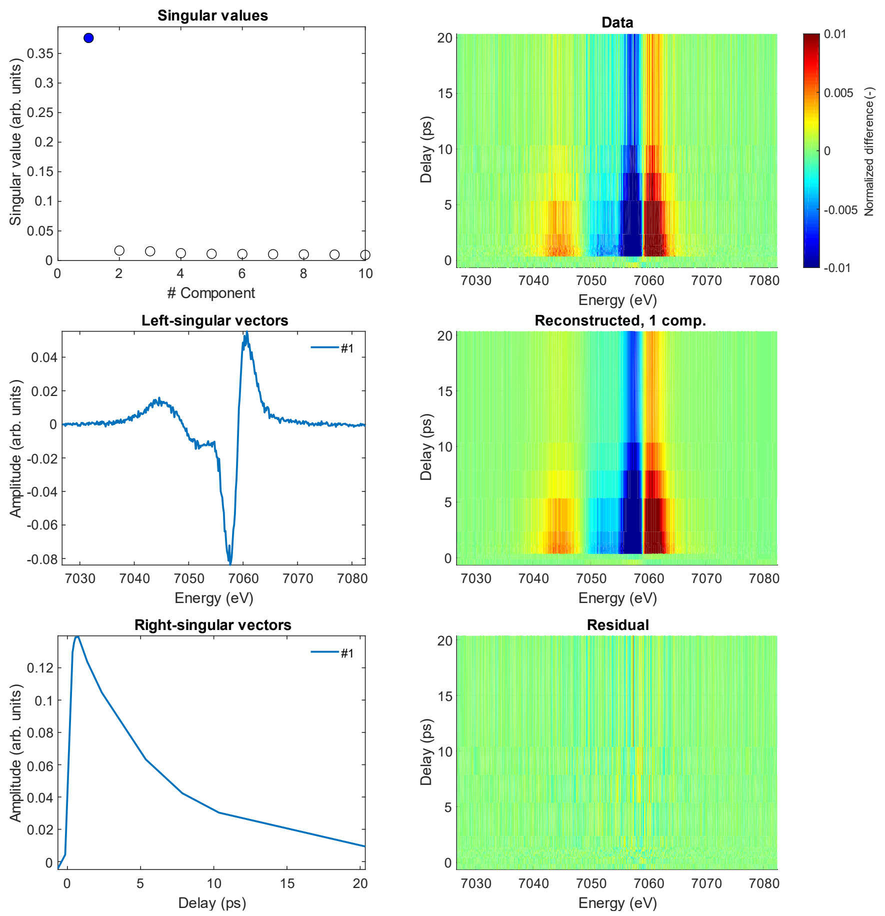

Supplementary Fig. 2. Singular value decomposition of the Kβ XES difference map in the -0.65 – 20 ps range with coarse step size. The left column shows the first ten singular values and the first left-/right-singular vectors. The right column shows the measured dataset, the reconstructed dataset using only the first left-/right-singular vectors and the residual.

Difference spectra averaged in the 0 – 0.2 ps, 0.6 – 0.85 ps and 2 – 3 ps ranges are shown in Supplementary Fig. 3a. Based on the reference spectra shown in Fig. 2a in the main text, at 7054 eV, the quintet difference signal with respect to the singlet is negative, the doublet and triplet difference signals are close to zero and the quartet difference signal is negative but has smaller magnitude than the quintet difference signal. The time-dependence of the quintet state is therefore approximated by the kinetic trace at 7054 eV ($k_{7054\,eV}$) and the quintet difference signal is subtracted off the total difference signal ΔI_{XES} according to:

$$\Delta I_{INT}(E,t) = \Delta I_{XES}(E,t) - k_{7054\,eV}(t) \cdot \Delta I_{Quintet}(E)$$

$k_{7054\,eV}$ is defined via integration of the noise-filtered Kβ XES difference signal (reconstruction of the XES map using four SVD components) in a narrow energy range around 7054 eV and normalized in the 0.6 – 0.85 ps range as shown in Supplementary Fig. 3b and the quintet spectrum $\Delta I_{Quintet}$ was extracted as the average difference spectrum of the noise-filtered dataset in the 0.6 – 0.85 ps range (Supplementary Fig. 3a). The extracted residual difference spectrum (Supplementary Fig. 3c) at 0.1 ps is then compared to doublet, triplet and quartet reference difference spectra, scaled by a fitted factor f_{INT} (Supplementary Fig. 3d-e). These model difference spectra have been generated by subtracting the $[Fe(2,2'\text{-bipyridine})_3]^{2+}$ electronic ground state Kβ XES reference spectrum ($I_{Singlet}$) from electronic ground state spectra of iron complexes with distinct electronic spin ground states (I_{INT}, Fig. 2a). We have used the $[Fe(2,2'\text{-bipyridine})_3]^{2+}$ spectrum, instead of the cyt *c* electronic ground state spectrum, because the higher concentration enables the spectrum to be measured with much higher signal-to-noise. While the residuals in Supplementary Fig. 3e qualitatively show that a triplet intermediate provides the best fit to the experimental data, further justification of the assignment of the intermediate to a ^{3}MC excited state merits discussion and ameliorates the limitations in using ground state spectra of distinct molecular complexes to model the electronic excited states of cyt *c*. Adjusted R^2 values (Supplementary Table 1) for the doublet, triplet and quartet references show that all three models fit the data reasonably well. We note that the fit exhibits some sensitivity to how $k_{7054\,eV}$ is chosen to subtract the quintet contribution from the difference spectra. We have repeated the fit procedure for different pixel ranges around 7054 eV used to define $k_{7054\,eV}$. The error bars shown in Supplementary Fig. 3b and Fig. 2d reflect the standard deviation of the time traces associated with individual pixels on the energy axis. In all cases, the triplet reference spectrum consistently fitted better than the doublet and quartet references.

	S = ½	S = 1	S = 3/2
f_{INT}	0.88	0.53	0.39
$\bar{R}^2$	0.891	0.935	0.924

Supplementary Table 1. Scaling factors f_{INT} and adjusted R^2 values ($\bar{R}^2$) for fitting the residual Kβ XES difference map (after subtraction of the quintet contribution) at 0.1 ps with different reference difference spectra.

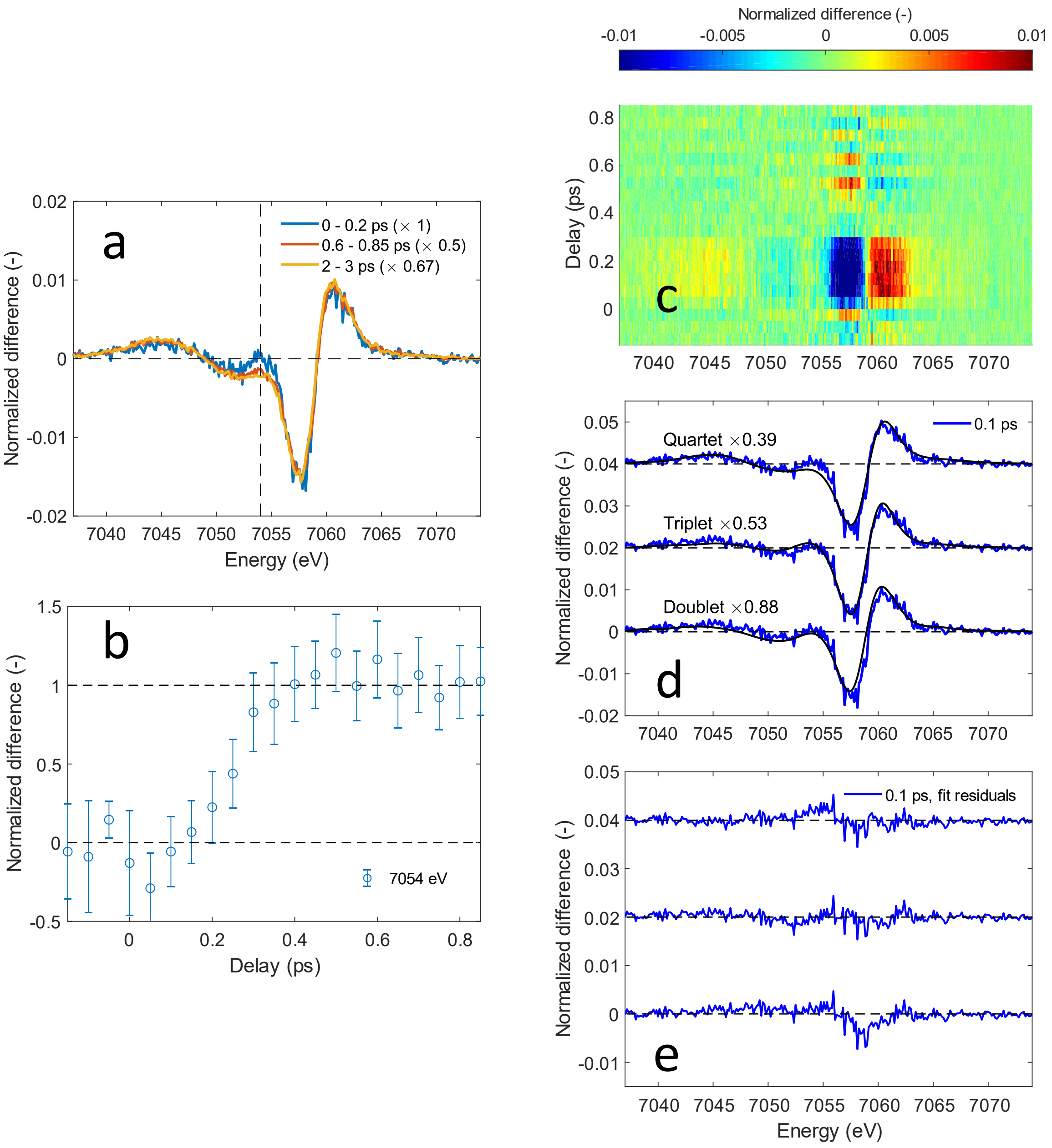

Supplementary Fig. 3. a Kβ XES difference spectra averaged in different time bins and scaled for comparison. **b** Kinetic trace of the Kβ XES difference map at 7054 eV. Error bars reflect the

standard deviation of the signal within a range of 7 detector pixels around this energy. Here, the analysis is based on the noise-filtered Kβ XES difference signal and therefore the kinetic trace exhibits smaller error bars than the kinetic trace shown in Fig. 2d for the same X-ray emission energy. **c** Kβ XES difference signal after subtraction of the quintet contribution. **d** Fits of the difference spectrum from **c** at 0.1 ps using a series of reference difference spectra. **e** Residuals of the fits shown in **d**.

Having validated the need for an intermediate electronic excited state in the relaxation from the $^1\pi$–π* state to the ^{5}MC state, we use the reference spectra shown in Fig. 2a to fit the time evolving XES difference spectra. Photoexcitation of the Q-band populates a $^1\pi$–π* excited state of the heme. The $^1\pi$–π* excited state then decays into an intermediate state (INT), which in turn feeds the longer-lived ^{5}MC excited state. Earlier studies reported a photodissociation quantum yield ≥80%[2] and we therefore approximate all processes to proceed with unity quantum yield. Moreover, we assume that despite the ultrafast nature of these processes, time-dependent populations can be approximated by solutions of a rate equation model. The following sequential four-level system considering the $^1\pi$–π*, INT, ^{5}MC and ground state (^{1}GS) is therefore analytically solved:

$$\dot{N}_{^1GS}(t) = \frac{1}{\tau_{^5MC}} \cdot N_{^5MC}(t)$$

$$\dot{N}_{^1\pi-\pi*}(t) = -\frac{1}{\tau_{^1\pi-\pi*}} \cdot N_{^1\pi-\pi*}(t)$$

$$\dot{N}_{INT}(t) = \frac{1}{\tau_{^1\pi-\pi*}} \cdot N_{^1\pi-\pi*}(t) - \frac{1}{\tau_{INT}} \cdot N_{INT}(t)$$

$$\dot{N}_{^5MC}(t) = \frac{1}{\tau_{INT}} \cdot N_{INT}(t) - \frac{1}{\tau_{^5MC}} \cdot N_{^5MC}(t)$$

The following initial conditions were used:

$$N_{^1GS}(t = t_0) = 1 - f_{exc}$$

$$N_{^1\pi-\pi*}(t = t_0) = f_{exc}$$

$$N_{INT}(t \leq t_0) = 0$$

$$N_{5_{MC}}(t \leq t_0) = 0$$

f_{exc} constitutes a lower bound for the photoexcitation yield since a photodissociation quantum yield of one was assumed for solving the rate equations. Resulting time-dependent populations are convoluted with a Gaussian instrument response function $\mathrm{IRF}(t) = \frac{1}{\sqrt{2\pi}\sigma} e^{-\frac{1}{2}\left(\frac{t-t_0}{\sigma}\right)^2}$. Our Kβ XES measurement does not have a spectroscopic signature for the $^1\pi$–π* excited state, so we use the exponential lifetime of $\tau_{1_{\pi-\pi*}} = 145 \pm 5$ fs measured by Bräm et al. for the $^1\pi$–π* excited state following direct excitation of the Q-band at 530 nm[3]. We use the 5.9 ps exponential lifetime for the ^{5}MC ($\tau_{5_{MC}}$) measured by Mara et al.,[1] leaving the lifetime of the intermediate state (τ_{INT}), f_{exc} as well as the $\mathrm{FWHM} = 2\sqrt{2\ln 2}\sigma$ and time zero (t_0) of the IRF, as the variables in the analysis. Depending on their spin state, the populations are represented by their respective reference spectrum and the best set of parameters is determined by fitting the calculated Kβ XES difference spectra to the experimental difference spectra in a least-squares procedure. The simulated difference signal ΔI_{sim} is calculated as

$$\Delta I_{sim}(E_i, t_j) = N_{INT} \otimes \mathrm{IRF}(t_j) \cdot \left(I_{INT}(E_i) - I_{\mathrm{Singlet}}(E_i)\right) + N_{5_{MC}} \otimes \mathrm{IRF}(t_j) \cdot \left(I_{5_{MC}}(E_i) - I_{\mathrm{Singlet}}(E_i)\right)$$

and the following RSS is minimized with respect to the fit variables:

$$\mathrm{RSS} = \sum_{i,j=1}^{n,m} \left(\Delta I_{exp}(E_i, t_j) - \Delta I_{sim}(E_i, t_j)\right)^2$$

The fit is evaluated in the -30 – 670 fs range. Supplementary Table 2 summarizes the set of parameters minimizing RSS for ^{2}MC, ^{3}MC and ^{4}MC intermediates. The error bounds were estimated based on a cutoff arbitrarily defined at a 3% increase in RSS. The differences in RSS between different models are relatively small. According to Burnham and Anderson,[4] a suitable way for model selection relies on utilizing the Akaike information criterion (AIC).[4, 5] Assuming normally distributed errors with a constant variance, the AIC value can be defined as AIC =

$n \log \hat{\sigma}^2 + 2K$, where $\hat{\sigma}^2$ is the estimated mean squared error, n is the number of time delay points and K is the total number of parameters. AIC values do not quantify in absolute terms how well a particular model fits the data but provide a formalism to sensitively compare models via differences of AIC values. We use the small sample corrected version, $\mathrm{AIC_c} = \mathrm{AIC} + \frac{2K(K+1)}{n-K-1}$. the resulting AIC differences $\Delta_i = \mathrm{AIC_{c,i}} - \mathrm{AIC_{c,min}}$ with respect to the best model from the given set are shown for each model i in Supplementary Table 2. These differences are then used to calculate the Akaike weights $w_i = e^{-\frac{1}{2}\Delta_i} / \sum_{r=1}^{R} e^{-\frac{1}{2}\Delta_r}$ (also shown in Supplementary Table 2) which reflect the relative likelihood of a model within the given set of R models. Based on established guidelines for comparing AIC differences and Akaike weights[4], we can reject the more complex ^{2}MC, ^{3}MC and ^{4}MC models including the $^1\pi$–π* lifetime as a fit parameter with high confidence. Among the simpler models with the $^1\pi$–π* lifetime fixed to 145 fs, the ^{3}MC model fits best but the AIC differences and Akaike weights do not allow us to clearly reject the ^{2}MC and ^{4}MC models.

INT	FWHM (fs)	t_0 (fs)	f_{exc} (-)	$\tau_{1_{\pi-\pi*}}$ (fs)	τ_{INT} (fs)	RSS	Δ_i	w_i
^{2}MC	99 ± 61	-15 ± 10	0.74 ± 2	145 (fixed)	70 ± 35	0.00278	0.94	0.27
^{2}MC	87 ± 55	-10 ± 9	0.78 ± 2	205 ± 26	<30	0.00272	7.13	0.01
^{3}MC	118 ± 61	-2 ± 9	0.74 ± 2	145 (fixed)	87 ± 51	0.00260	0	0.43
^{3}MC	71 ± 47	-14 ± 9	0.76 ± 2	179 ± 24	56 ± 72	0.00261	6.55	0.02
^{4}MC	179 ± 58	24 ± 10	0.74 ± 2	145 (fixed)	<50	0.00280	1.04	0.26
^{4}MC	106 ± 59	-2 ± 10	0.78 ± 2	205 ± 25	<43	0.00273	7.18	0.01

Supplementary Table 2. Fitted variables, RSS values, AIC differences (Δ_i) and Akaike weights (w_i) for doublet, triplet and quartet intermediates either with fixed or fitted $\tau_{1_{\pi-\pi*}}$.

Assigning INT to a ^{3}MC represented by the triplet reference spectrum therefore provides the best agreement between the model and the experiment, consistent with the previous analysis (Supplementary Table 1 and Supplementary Fig. 3d-e) limited to the difference spectrum at 0.1 ps after subtraction of the ^{5}MC contribution. Based on this analysis, we favor the sequential ^{3}MC model. The best fit gives an instrument response function FWHM of 118 ± 61 fs, in excellent agreement with an expected FWHM of ~110-130 fs based on the optical and X-ray pulse durations (~50 fs each) and the group velocity mismatch between the X-ray and optical pulses (on the order of 1.1 fs/µm calculated with an index of refraction of 1.33 for water) leading to a time delay spread of roughly 83-110 fs in the 75–100 µm liquid sample jet.[6] The triplet lifetime is fitted to 87 ± 51 fs and the fraction of ^{5}MC species is fitted to 74 ± 2 %.

Supplementary Note 2: Power dependence of the transient signal

The presence of multi-photon absorption effects due to high pump laser fluences can distort the observed excited-state dynamics. For this reason, power titration scans were performed during both, the first XES and the second combined XES and XSS experiments. For the first experiment, we have discussed pump fluence controls in the Supplementary Information of Mara et al.[1] Despite the high excitation yield, we have been able to demonstrate that the observed dynamics conform to those measured under more benign excitation conditions, where direct comparison can be made. For the second X-ray experiment, the same excitation fluence was used as for the first experiment.

Supplementary Note 3: Picosecond kinetics and heat dissipation analysis

As discussed in the main text, the XSS difference signal in the Q = 0.25 – 1.2 $Å^{-1}$ range is due to the protein structural response. After ~2 ps, this difference signal can be well approximated by a single SVD component (Supplementary Fig. 4a-b) and the resulting right-singular vector is exponentially fitted. The resulting time constant of 5.2 ± 1 ps agrees with the ^{5}MC decay timescale previously determined from Kβ XES.[1] After ~3 ps, the magnitude of the protein difference signal in the Q = 1.3 – 3.2 $Å^{-1}$ range is negligible compared to the difference signal of the photoinduced solvent response. The water excess energy is therefore quantified by fitting the XSS difference signal ΔS in the Q = 1.3 – 3.2 $Å^{-1}$ range with the well-known bulk water heat differential $\left.\frac{\partial S(Q)}{\partial T}\right|_{\rho}$:[7, 8]

$$\Delta S(Q,t) \approx \left.\frac{\partial S(Q)}{\partial T}\right|_{\rho} \cdot \Delta T(t)$$

The water density change occurs on a slower timescale and is neglected in the fit. A comparison of the experimental and fitted difference scattering curves in the 3 – 14 ps range is shown in Supplementary Fig. 4c. The increase in bulk water temperature ΔT occurs due to energy dissipation from the protein and reaches ~0.17 K at 14 ps. Using the specific heat at constant volume $C_V \sim 74.54\ \mathrm{J \cdot mol^{-1} \cdot K^{-1}}$ and the number of water molecules per liquid unit cell $\frac{c_{H_2O}}{c_{cyt\,c}}$, ΔT is converted to the bulk water excess energy shown in Supplementary Fig. 4d (blue circles). It has an exponentially fitted rise time of 7.0 ± 1.1 ps. The protein excess energy (red circles in Supplementary Fig. 4d) is estimated by subtracting the water excess energy from the mean energy initially deposited in a cyt *c* molecule through the photon absorption process. Photoexcitation at 520 nm deposits ~2.4 eV at the heme. The estimated excitation yield is ~0.74 as determined from the ^{5}MC signature in the Kβ XES assuming a dissociation quantum yield $\phi_{dissoc} \approx 1$.[2] Therefore, the initially deposited energy in a cyt *c* molecule is $\approx 2.8 \cdot 10^{-19}$ J. This corresponds well with the amount of energy released to the water after 14 ps ($\sim 3.0 \cdot 10^{-19}$ J, Supplementary Fig. 4d). Therefore, the protein reaches thermal equilibrium with the water bath with an exponential time constant of ~7 ps.

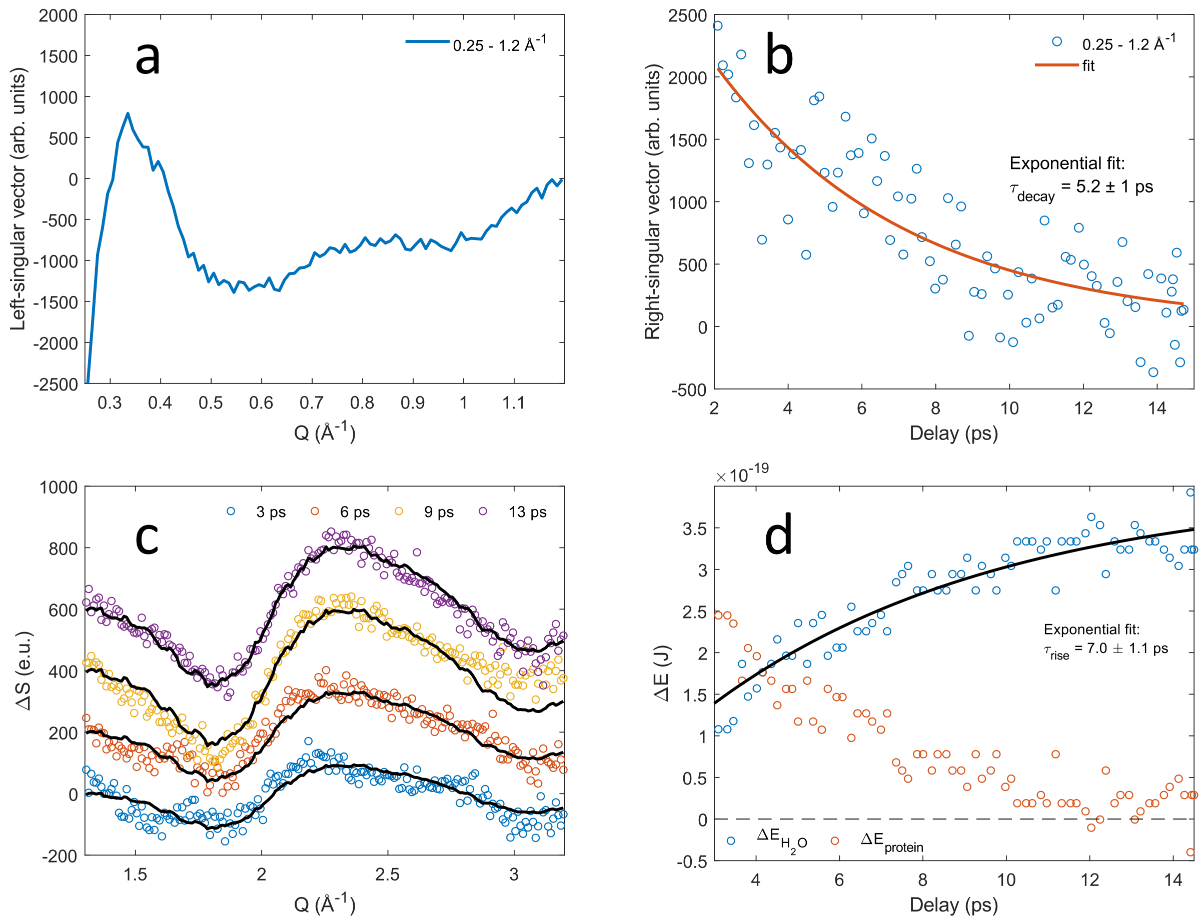

Supplementary Fig. 4. a-b First left- and right-singular vectors of the XSS difference signal in the Q = 0.25 – 1.2 Å^{-1} and 2 – 14.5 ps ranges reflecting the protein structural response. Fit results of the bulk water heating response in the Q = 1.3 – 3.2 Å^{-1} range are shown in **c** and the time evolution of the bulk water (blue circle) and protein (red circle) excess energies is shown in **d**.

Supplementary Note 4: Structural analysis of the sub-picosecond XSS data

Measured total scattering curves are dominated by the water peak and the solute contribution can be neglected due to the large solvent to solute concentration ratio ($c_{H_2O} = 55.5$ M, $c_{cyt\,c}$~3.5 mM). The data are therefore rescaled such that the mean total scattering signal of a single water molecule in solution in the 1 – 3 Å^{-1} range is 41.3 e.u., then multiplied by $\frac{c_{H_2O}}{c_{cyt\,c}}$ to reflect a single liquid unit cell containing one cyt *c* unit and $\frac{c_{H_2O}}{c_{cyt\,c}}$ water molecules. The same normalization factor is then applied to the experimental difference scattering curves which exhibit features due to changes in both, protein structural evolution and solvent heating. Structural analysis is performed by fitting the noise-filtered difference scattering curves (shown in Fig. 4) at each time delay using simulated total scattering curves parameterized via coordinates of the Met80 and His18 axial ligands and the bulk water heating contribution.[7, 8] For the ground state structure, cyt *c* solution phase coordinates are directly taken from the PDB database structure 2GIW[9]. These coordinates are then used as a starting point to parameterize excited state structures. Each protein structure contains 866 atoms (ignoring hydrogen atoms) and their orientationally averaged total scattering curve is calculated[10] using the Debye scattering equation:

$$S(Q) = \sum_{i=1}^{N} |f_i(Q)|^2 + 2 \sum_{i=1}^{N-1} \sum_{j=i+1}^{N} f_i(Q) f_j^*(Q) \frac{\sin Qr_{ij}}{Qr_{ij}}$$

The scattering Q-vector is related to the scattering angle via $Q = \frac{4\pi}{12.398/E} \sin\frac{\theta}{2}$, with $E = 8$ keV, the incident energy of the X-rays. f_i, the non-dispersive part of the atomic scattering factor is approximated for each element as $f_i(k) = c + \sum_{i=1}^{4} a_i e^{-b_i\left(\frac{Q}{4\pi}\right)^2}$ with the Cromer-Mann coefficients a_i, b_i, and c as tabulated.[11] The parameterization of excited state structures focused on the His18 and Met80 axial ligand positions. In detail, the imidazole ring of the histidine (atoms #272–276 of PDB structure 2GIW) was translated along the Fe(II)-N(His18) axis and the Met80 residue (atoms #1247–1254 and #1264) was rotated around an axis defined by atoms #1247 (N) and #1249 (C), effectively changing the Fe(II)-S(Met80) distance as shown in Fig. 4d in the main text. To support the assumption that the dominant effect on the observed XSS difference signal stems from the axial ligand coordinates, we have also implemented models approximating the Fe

out-of-plane motion and the Cys14 (atoms #220-225 of PDB structure 2GIW) and Cys17 (atoms #257-262) positions. The Fe out-of-plane motion was parameterized by translating the Fe atom along the Fe(II)-N(His18) axis. The Cys14 and Cys17 movements resulting from photoexcitation at the heme are difficult to infer and therefore constrained to a translation along a vector defined by the position of the Fe atom and the average position of the atoms of the residue. Fe(II)-Met80 and Fe(II)-His18 distances, the displacement of the Fe-atom from its original position towards N(His18) and Fe(II)-Cys14 and Fe(II)-Cys17 distances thus define the set of structural parameters $\vec{\mathbf{a}}$ to be optimized alongside the amplitude of the bulk water heat signal f_{H2O}. Difference scattering curves $\Delta S(Q,\vec{\mathbf{a}})_{solute} = S_{ES}(Q,\vec{\mathbf{a}}) - S_{GS}(Q)$ are then calculated for the parameter ranges indicated in Supplementary Table 3. At each time delay t_j, $\vec{\mathbf{a}}$ and f_{H2O} were chosen to minimize the RSS in the Q-range 0.265 – 3.325 Å^{-1}:

$$\mathrm{RSS}(t_j,\vec{\mathbf{a}}, f_{H2O}) = \sum_{i=1}^{N_Q}\Big(\Delta S_{exp}(Q_i,t_j) - f_{ES}(t)\cdot\Delta S_{solute}(Q_i,\vec{\mathbf{a}}) - f_{H2O}\cdot\Delta S_{H2O}(Q_i)\Big)^2$$

As shown in Supplementary Note 3, the measured XSS difference signal around 15 ps agrees well with the scaled bulk water heat differential.[7, 8] It is therefore directly chosen to represent the bulk water heating contribution ΔS_{H2O}. The fitted scaling factor f_{H2O} is proportional to the increase in bulk water temperature. Changes in bulk water density occur on a slower timescale and are not considered in the fit. The time-dependent excited state population $f_{ES}(t)$ is approximated by a constant in the 0.06 – 0.3 ps range and fixed to $f_{exc} = 0.74$, the value derived from the XES analysis. To compare different solute structural models using an F-test,[12] RSS values are evaluated for delays in the 0.06 – 0.14 ps range and then averaged. The lower delay time limit reflects limitations in modeling the IRF. RSS values and the number of fit parameters are summarized in Supplementary Table 4 for different models labeled as A–F. Models A-C focus on the Met80 and His18 positions while models D-F consider either the Fe out-of-plane, Cys14 or Cys17 motions in addition to the axial ligand positions. Within the 0.06 – 0.14 ps range, the fits for models D-F retain the Fe atom, Cys14 and Cys17 positions very close to their ground state positions. The addition of any of these structural parameters does therefore not significantly decrease the RSS of the fit. A comparison between experimental and simulated curves for models A-F is shown in Supplementary Fig. 5.

Fit parameters	Ground state value (Å)	Optimization range (Å)
Fe(II)-S(Met80)	2.38	$2.31 - 3.09$
Fe(II)-N(His18)	1.94	$1.84 - 2.44$
Fe out-of-plane motion	0.00	$-0.10 - 0.30$
Fe(II)-Cys14	0.00	$-0.045 - 0.09$
Fe(II)-Cys17	0.00	$-0.045 - 0.09$

Supplementary Table 3. Optimization ranges for fit parameters. Fe out-of-plane, Fe(II)-Cys14 and Fe(II)-Cys17 motions are relative to the ground state.

Model	Fit parameters	p	$\overline{\mathrm{RSS}}$	$\overline{R^2}$
A	Fe(II)-S(Met80), f_{H2O}	2	$4.14 \cdot 10^5$	0.794
B	Fe(II)-N(His18), f_{H2O}	2	$10.21 \cdot 10^5$	0.493
C	Fe(II)-S(Met80), Fe(II)-N(His18), f_{H2O}	3	$3.23 \cdot 10^5$	0.840
D	Fe(II)-S(Met80), Fe(II)-N(His18), Fe out-of-plane, f_{H2O}	4	$2.96 \cdot 10^5$	0.851
E	Fe(II)-S(Met80), Fe(II)-N(His18), Fe(II)-Cys14, f_{H2O}	4	$3.22 \cdot 10^5$	0.841
F	Fe(II)-S(Met80), Fe(II)-N(His18), Fe(II)-Cys17, f_{H2O}	4	$3.19 \cdot 10^5$	0.842

Supplementary Table 4. Comparison of time-averaged best fit RSS- and R^2 values for different models. p is the number of fit parameters and $\overline{\text{RSS}}$ and $\overline{R^2}$ are evaluated in the range $60\ fs < t_i \leq 140\ fs$.

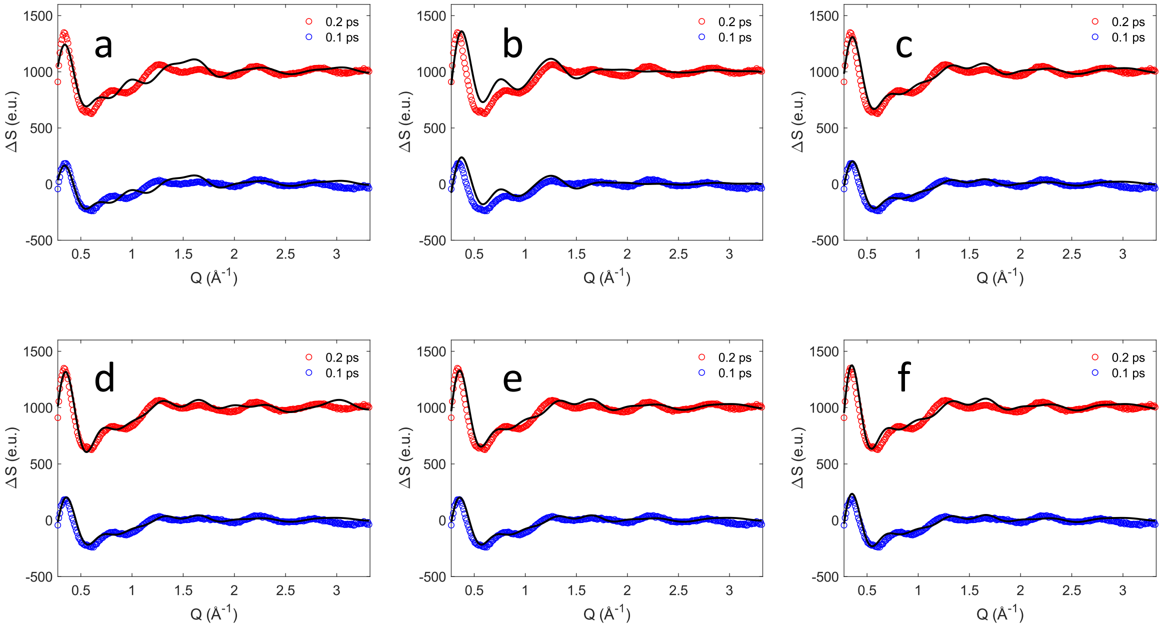

Supplementary Fig. 5. XSS difference signal fits for two different time delays using parameter sets as defined in Supplementary Table 4. **a**-**f** represent models A-F.

To compare two models with different numbers of fit parameters p_1 and p_2, F statistic is calculated using $F = \frac{(\text{RSS}_1 - \text{RSS}_2)(n - p_2)}{\text{RSS}_2(p_2 - p_1)}$. n is the number of 'relevant independent data points' and can be estimated either by counting the number of components above the noise in power spectra of difference scattering curves, or from the minimum order of a polynomial fit of the difference scattering curve.[10] Both methods indicate $n \sim 19$ around 0.1 ps (Supplementary Fig. 6). The model with the larger number of parameters is rejected if the evaluated F-value is below a certain threshold. For 5% significance level, the relevant F-distribution upper critical values

$F_{.05}(p_2 - p_1, n - p_2)$ are $F_{.05}(1,16) = 4.494$ and $F_{.05}(1,15) = 4.543$ for comparing models A-B with C and C with D-F, respectively. The estimated F-values are $F^{AC}{\sim}4.51$, $F^{BC}{\sim}34.58$, $F^{CD}{\sim}1.37$, $F^{CE}{\sim}0.05$ and $F^{CF}{\sim}0.19$ thus indicating that within the constraints of the structural models, elongation of both axial ligands (model C) is necessary but no additional Fe out-of-plane motion (model D), Cys14 (model E) or Cys17 (model F) translation is required for a satisfactory fit of the data. We note that our simplified parameterization of the Fe out-of-plane motion does not adequately describe the heme core expansion and doming. Analogously, models E-F may not accurately parameterize the structural response of the Cys14 and Cys17 residues to the photoexcitation process. However, as described in the main text, structural changes in the Cys14 and Cys17 residues are expected to be less significant at the earliest delays. The absence of a significant improvement in fit quality when using models D-F therefore provides qualitative support for the simpler models focused on changes in axial ligand coordinates. The time-averaged R^2 values also shown in Supplementary Table 4 further confirm that model C captures a significant part of the variation in the XSS difference signal without invoking additional degrees of freedom.

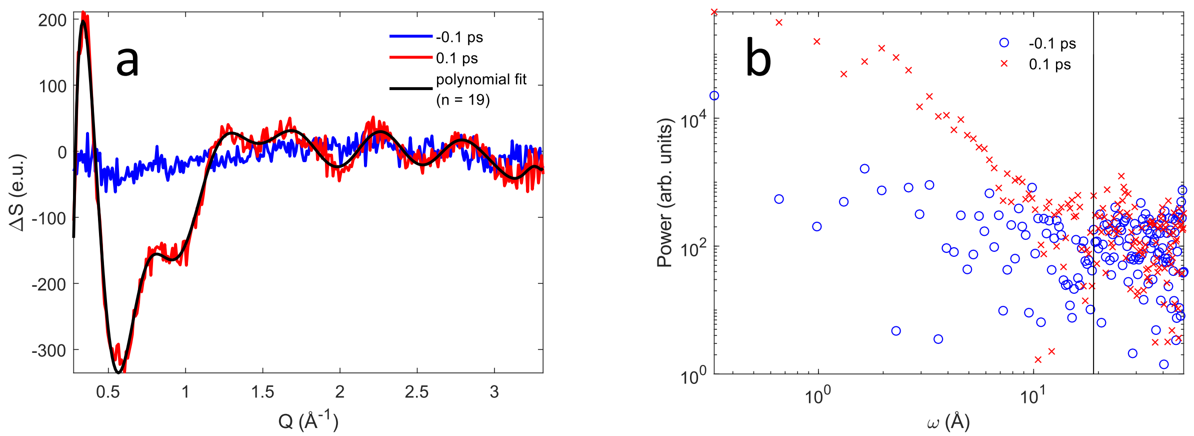

Supplementary Fig. 6. a Nineteenth-order polynomial fit of the difference scattering signal at 0.1 ps. **b** Power spectrum $S(\omega) = |\mathcal{F}(\Delta S)|^2$ calculated from the Fourier transforms at -0.1 ps (blue circles) and 0.1 ps (red crosses). The vertical line is at $\omega = 19$ Å above which the two spectra overlap.

Using Fe K-edge XANES, we have previously reported Fe coordination shell distances for the ^{5}MC state concluding that at 600 fs, Fe(II)-S(Met80) > 2.90 Å and the Fe atom has moved out of the heme plane.[1] A direct comparison of these previous results with the ones presented in this study is not attempted for the following reasons: Fe K-edge XANES is a local structural probe only sensitive to the photoactive site around the Fe atom. The shape resonances appear to be dominated by heme distortions including the Fe out-of-plane motion while changes in the Fe(II)-S(Met80) distance predominantly influence the rising edge and edge peak regions but to a lesser extent the shape resonances. In contrast, XSS is a global structural probe sensitive to the entire ensemble of protein and solvent atoms. The analysis based on a simplistic model of the photoactive site structure presented in this study is only reasonable within the first ~300 fs where structural changes in the tertiary protein structure are less important. Even though the chosen local coordinates do not fully capture the active site structural rearrangement upon photoexcitation, they demonstrate that moving the Met80 and His18 axial ligands away from the heme captures the main characteristics of the observed changes in the scattering signal. Within these constraints, we find Fe(II)-S(Met80) <2.65 Å indicating negligible Fe out-of-plane motion. The Fe atom position relative to S(Met80) may be further influenced by photoinduced heme distortions neglected in this analysis. Moreover, the Fe(II)-S(Met80) distance likely increases on the ~300 – 600 fs timescale due to delayed activation of the Fe out-of-plane motion observed at 600 fs by the Fe K-edge XANES results. This is supported by the delayed activation of certain structural degrees of freedom illustrated in Fig. 4 in the main text.

It is noted that the 2GIW cyt *c* active site ground state structure[9] used in the present XSS analysis differs somewhat from the local ground state structure previously reported in the Fe K-edge XANES study. We have therefore fitted the previously reported experimental Fe K-edge XANES using MXAN with the 2GIW structure. Despite the somewhat longer Fe(II)-S(Met80) bond (Supplementary Table 3), the agreement is satisfactory (Supplementary Fig. 7).

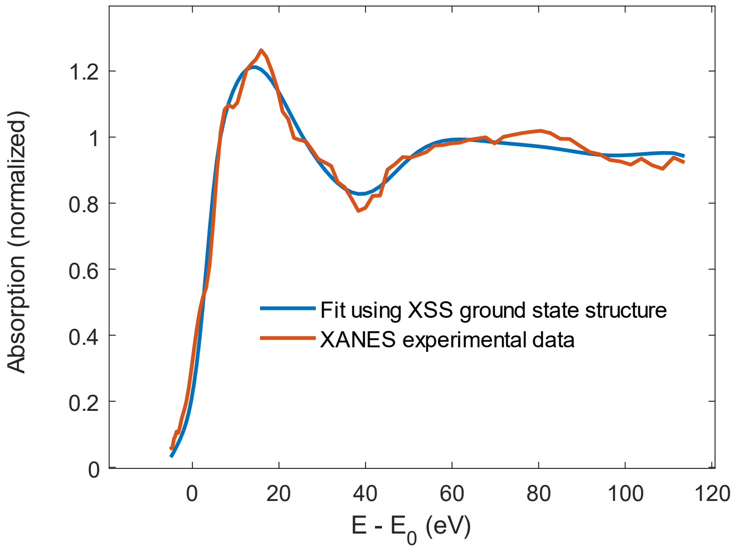

Supplementary Fig. 7. Comparison of the MXAN fit using the 2GIW active site ground state structure used in the XSS analysis and the XANES experimental data from Mara et al.[1]

Supplementary Note 5: DFT calculations

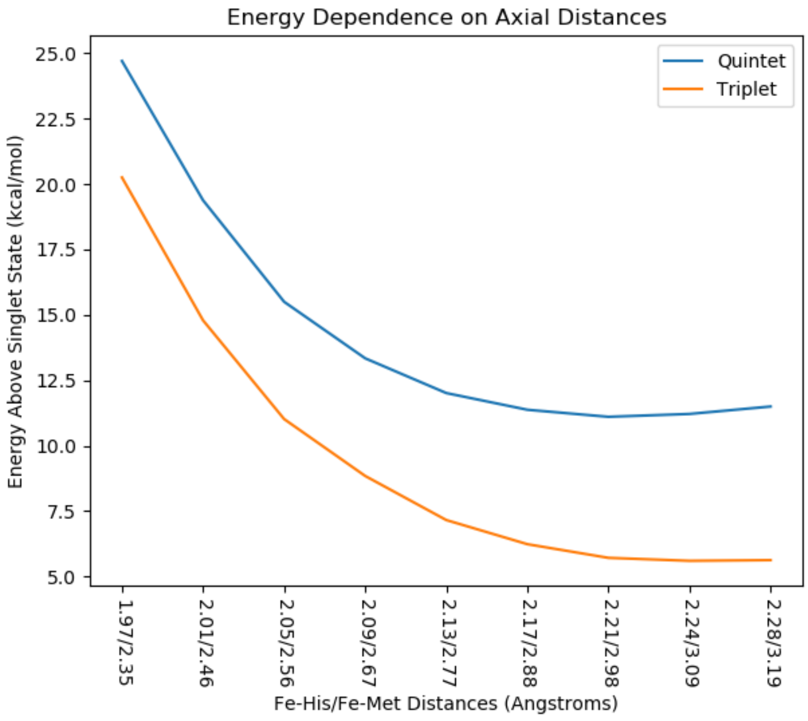

Supplementary Fig. 8. Triplet and quintet energy dependence on the axial ligand lengths. Both Fe(II)-His18 and Fe(II)-Met80 distances were elongated iteratively between their singlet distance and the relaxed triplet distance, and single point energy calculations were performed at each structure. Quintet surface is at least 5 kcal/mol above the triplet and does not cross with only axial elongation.

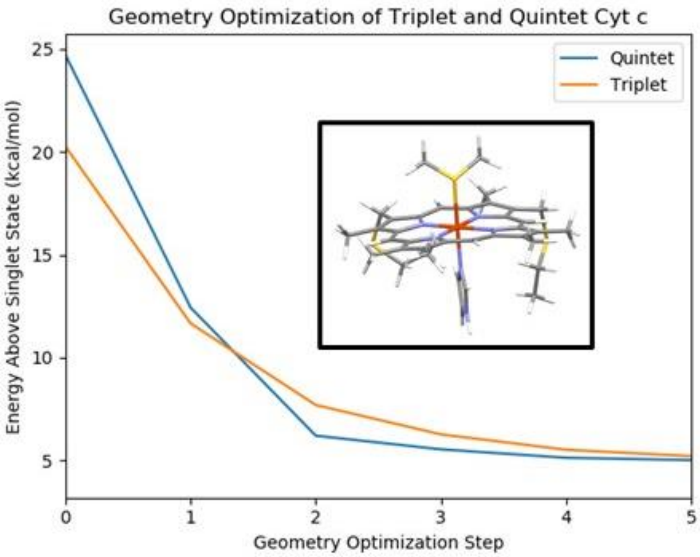

Supplementary Fig. 9. Geometry optimization of the triplet and quintet species, beginning from the optimized singlet state. Triplet and quintet surfaces cross between the 1st and 2nd optimization steps, ~ 12 kcal/mol above the singlet state energy. Structure of the triplet species at step 1 is shown in black box. Both the triplet and quintet species proceed towards relaxed structures ~ 4 kcal/mol above the singlet structure energy.

Structure	Energy (kcal/mol)	Fe(II)-N(His18) (Å)	Fe(II)-S(Met80) (Å)	Fe(II)-N(Por) (Å)	N(Por)-N(Por) (Å)
Singlet	0.0	1.97	2.35	2.00	4.01
Triplet	20.2	2.29	3.06	2.00	4.00
Quintet	24.7	2.21	2.96	2.08	4.15
Triplet (Crossing Point)	11.7	2.08	2.47	2.00/2.03	4.00/4.06
Quintet (Crossing Point)	12.4	2.06	2.44	2.04	4.09

Supplementary Table 5. Relevant parameters for DFT structures.

Fe	-0.868	-0.07614	-0.33092
C	-4.10998	0.78437	0.28201
C	-1.62071	-3.34549	0.26638
C	2.28958	-0.96338	-1.25027
C	-0.0273	3.2129	-0.55743
N	-2.53413	-1.08148	0.15328
C	-3.78838	-0.56724	0.34434
C	-4.7405	-1.616	0.67522
C	-4.02918	-2.78374	0.69646
C	-2.65786	-2.42915	0.36637
C	-4.50529	-4.17297	0.98242
C	-6.19788	-1.39398	0.93055
N	0.14285	-1.80571	-0.45108
C	-0.31378	-3.05219	-0.11205
C	0.72919	-4.04892	-0.2469
C	1.83905	-3.38098	-0.69641
C	1.44917	-1.98509	-0.82119
C	0.58542	-5.50396	0.06639
C	3.1985	-3.93616	-0.96519
N	0.78729	0.92689	-0.84081
C	1.99208	0.39523	-1.22499
C	2.96725	1.43495	-1.47912
C	2.33932	2.62007	-1.19261
C	0.97469	2.28241	-0.82311
C	4.38845	1.22344	-1.89261
C	2.95685	3.97608	-1.14679
N	-1.87437	1.64864	-0.20331
C	-1.35728	2.91467	-0.2947
C	-2.39387	3.9173	-0.10612
C	-3.55764	3.22905	0.10067
C	-3.20911	1.81818	0.05002
C	-2.16761	5.39562	-0.14989
C	-4.93642	3.7581	0.34059
H	-5.14763	1.05896	0.46289
H	-1.8534	-4.38668	0.48223
H	3.28868	-1.23771	-1.57974
H	0.24353	4.26633	-0.58783
H	-4.36169	-4.83833	0.1181
H	-5.57303	-4.18687	1.2323
H	-3.96379	-4.62296	1.82778
H	-6.70266	-0.96468	0.05245
H	-6.70935	-2.33253	1.17609
H	1.48704	-6.06098	-0.21294

H	-0.2675	-5.95175	-0.46453
H	0.42456	-5.67207	1.14145
H	3.7991	-3.25863	-1.58253
H	4.8225	2.14454	-2.30107
H	4.47785	0.44469	-2.66277
H	5.02056	0.92497	-1.04307
H	2.20814	4.77558	-1.18987
H	-3.09888	5.94892	0.02079
H	-1.76807	5.71795	-1.12291
H	-1.4478	5.72025	0.61631
H	-5.33225	3.44056	1.317
H	-4.95399	4.85447	0.32172
C	-0.79264	1.05313	-3.56187
S	-1.63318	-0.19783	-2.55583
C	-1.01553	-1.69402	-3.37298
H	-1.15071	1.00158	-4.59664
H	0.29446	0.92448	-3.52113
H	-1.43055	-2.54888	-2.82975
H	0.0779	-1.74236	-3.32727
H	-1.36098	-1.71973	-4.41306
N	-0.3696	0.07051	1.57162
C	-1.19639	0.32819	2.56698
C	0.88476	-0.06202	2.12312
N	-0.52075	0.36608	3.74251
C	0.80979	0.11914	3.47893
S	4.03897	4.25106	0.33166
C	2.87144	3.84902	1.67507
C	3.60792	3.63678	3.00556
S	4.17662	-4.4151	0.53077
C	4.19011	-2.87827	1.51158
C	5.11531	-1.766	0.96013
H	-2.26184	0.48938	2.46877
H	1.74509	-0.27672	1.50366
H	2.1333	4.65606	1.77262
H	2.3362	2.93282	1.39804
H	2.88112	3.39946	3.79385
H	4.15974	4.53499	3.31245
H	4.32192	2.80695	2.93088
H	3.15569	-2.52358	1.61219
H	4.5256	-3.19662	2.50713
H	5.09211	-0.8888	1.6225
H	4.79413	-1.44151	-0.03462
H	6.14932	-2.12487	0.88955
H	-0.92911	0.54219	4.64926

H	-1.04964	2.02786	-3.13524
H	-5.64478	3.40487	-0.42329
H	3.64198	4.13353	-1.99074
H	3.13475	-4.88482	-1.51572
H	1.5588	0.09098	4.25792
H	-6.36529	-0.70044	1.76826

Supplementary Table 6. Coordinates of DFT optimized singlet structure.

Fe	-0.87584	0.06302	-0.24713
C	-3.94812	1.37695	0.38491
C	-2.11398	-3.08405	0.25747
C	2.09576	-1.26188	-1.23573
C	0.41385	3.21129	-0.55396
N	-2.66506	-0.70038	0.21291
C	-3.82945	-0.00642	0.41934
C	-4.92822	-0.90826	0.71947
C	-4.40466	-2.17249	0.70339
C	-2.99684	-2.0226	0.38097
C	-5.08845	-3.48091	0.94602
C	-6.33716	-0.47773	0.9801
N	-0.14858	-1.80234	-0.4385
C	-0.77819	-2.97709	-0.11705
C	0.11576	-4.10482	-0.25257
C	1.31372	-3.59056	-0.68438
C	1.12318	-2.15717	-0.80404
C	-0.22969	-5.52905	0.04521
C	2.58837	-4.32732	-0.93182
N	0.88448	0.82707	-0.81312
C	1.99286	0.12322	-1.218
C	3.09606	1.01102	-1.51291
C	2.65042	2.279	-1.2367
C	1.26287	2.14538	-0.83199
C	4.4591	0.59407	-1.96454
C	3.45265	3.53588	-1.23904
N	-1.61777	1.91809	-0.12513
C	-0.93597	3.10067	-0.2525
C	-1.81752	4.23639	-0.05617
C	-3.06059	3.71486	0.18457
C	-2.91167	2.27165	0.14619
C	-1.39442	5.66971	-0.1294
C	-4.34785	4.43269	0.44151
H	-4.9336	1.79691	0.57698
H	-2.49992	-4.08295	0.45017

H	3.04043	-1.67463	-1.58044
H	0.82737	4.21571	-0.60965
H	-5.03593	-4.13771	0.0651
H	-6.14926	-3.33984	1.18552
H	-4.63288	-4.02952	1.78385
H	-6.76302	0.05308	0.11606
H	-6.98601	-1.33629	1.19048
H	0.56803	-6.20787	-0.2782
H	-1.15699	-5.83601	-0.46001
H	-0.3741	-5.69469	1.12302
H	3.2819	-3.74706	-1.55068
H	5.02184	1.44658	-2.36384
H	4.41053	-0.17087	-2.75204
H	5.05418	0.18044	-1.13694
H	2.82028	4.43087	-1.26594
H	-2.23626	6.34515	0.06465
H	-0.99115	5.92615	-1.12032
H	-0.61149	5.90217	0.60772
H	-4.77318	4.16799	1.42087
H	-4.21041	5.52048	0.42697
C	-0.41576	0.8878	-4.02084
S	-1.76581	0.01829	-3.17866
C	-1.37748	-1.6866	-3.65947
H	-0.4859	0.77321	-5.1094
H	0.55855	0.53401	-3.6631
H	-2.1159	-2.32983	-3.16874
H	-0.37976	-1.97209	-3.30584
H	-1.45059	-1.82078	-4.74556
N	-0.24698	0.08725	1.95575
C	-1.06451	0.10161	2.98489
C	1.02518	0.07176	2.47366
N	-0.37072	0.09769	4.15419
C	0.97264	0.07643	3.84466
S	4.62236	3.67245	0.18953
C	3.46161	3.48587	1.58478
C	4.20941	3.18484	2.89161
S	3.47415	-4.91017	0.58229
C	3.74192	-3.36531	1.5154
C	4.80202	-2.41485	0.90707
H	-2.14597	0.1154	2.92548
H	1.89019	0.05463	1.82205
H	2.8646	4.40135	1.69002
H	2.7765	2.662	1.35134
H	3.48734	3.0795	3.71211

H	4.90717	3.99065	3.15454
H	4.78147	2.25198	2.80986
H	2.77693	-2.85525	1.63265
H	4.05726	-3.70508	2.5104
H	4.91458	-1.52152	1.53772
H	4.5077	-2.08178	-0.09273
H	5.77464	-2.91647	0.83241
H	-0.76949	0.1047	5.08274
H	-0.51461	1.94846	-3.76581
H	-5.10588	4.18673	-0.31657
H	4.11557	3.58773	-2.11311
H	2.40105	-5.26622	-1.47063
H	1.73985	0.06365	4.60685
H	-6.40369	0.20375	1.84123

Supplementary Table 7. Coordinates of DFT optimized triplet structure.

Fe	-0.86347	0.0021	-0.2266
C	-4.01379	1.16904	0.4698
C	-1.98574	-3.21775	0.14385
C	2.18885	-1.1662	-1.25517
C	0.25466	3.2279	-0.58982
N	-2.67899	-0.87249	0.24799
C	-3.85617	-0.22113	0.47848
C	-4.91802	-1.1891	0.74769
C	-4.34209	-2.42934	0.66477
C	-2.93366	-2.21174	0.34071
C	-4.97745	-3.77241	0.83906
C	-6.34204	-0.83205	1.03524
N	-0.04434	-1.88033	-0.54263
C	-0.6397	-3.07069	-0.23706
C	0.31927	-4.14868	-0.36924
C	1.50816	-3.56714	-0.76022
C	1.25343	-2.14174	-0.87114
C	0.06134	-5.5945	-0.08734
C	2.82172	-4.24194	-0.97292
N	0.90171	0.88494	-0.86464
C	2.04238	0.22427	-1.2315
C	3.11018	1.17747	-1.4925
C	2.58645	2.4212	-1.23583
C	1.18741	2.21947	-0.8669
C	4.50765	0.83403	-1.89899
C	3.32601	3.71601	-1.20446

N	-1.73735	1.87826	-0.111
C	-1.09998	3.07547	-0.26123
C	-2.0383	4.16363	-0.03369
C	-3.25045	3.58044	0.25362
C	-3.03686	2.14253	0.20687
C	-1.69807	5.61869	-0.117
C	-4.55821	4.24146	0.55813
H	-5.01351	1.5416	0.68958
H	-2.33019	-4.23943	0.29884
H	3.164	-1.53091	-1.57111
H	0.61544	4.2537	-0.64914
H	-4.90504	-4.37534	-0.07815
H	-6.04114	-3.68616	1.09158
H	-4.49412	-4.34947	1.64155
H	-6.79944	-0.28935	0.19473
H	-6.95214	-1.72438	1.22041
H	0.80302	-6.23596	-0.57962
H	-0.93258	-5.90616	-0.43667
H	0.1171	-5.81243	0.98952
H	3.50129	-3.62893	-1.57614
H	5.0518	1.72121	-2.24457
H	4.52216	0.09522	-2.71296
H	5.08482	0.41304	-1.06227
H	2.64925	4.57781	-1.24119
H	-2.56734	6.24963	0.10408
H	-1.33901	5.89501	-1.11942
H	-0.90516	5.89182	0.59505
H	-4.94295	3.94768	1.54603
H	-4.46806	5.33439	0.55423
C	-0.89123	1.34226	-3.79449
S	-1.81183	-0.01721	-3.02685
C	-0.95438	-1.42615	-3.78113
H	-1.03788	1.35433	-4.88121
H	0.17569	1.27195	-3.55356
H	-1.3891	-2.33241	-3.34659
H	0.11487	-1.40394	-3.54011
H	-1.10058	-1.44105	-4.86794
N	-0.20063	0.02007	1.88278
C	-1.00264	-0.03412	2.92485
C	1.08075	0.06822	2.37796
N	-0.28918	-0.02103	4.07866
C	1.04892	0.04244	3.74822
S	4.4413	3.89376	0.26368
C	3.24984	3.61277	1.61656

C	3.96685	3.33441	2.94549
S	3.69856	-4.77495	0.56683
C	3.84804	-3.21743	1.50462
C	4.88281	-2.21386	0.93954
H	-2.08345	-0.08347	2.87846
H	1.9326	0.1142	1.71104
H	2.59424	4.4881	1.71457
H	2.62382	2.75368	1.3473
H	3.22256	3.16815	3.73554
H	4.60183	4.17711	3.24879
H	4.59989	2.44111	2.87023
H	2.85364	-2.75785	1.57762
H	4.1369	-3.53784	2.51398
H	4.91974	-1.31342	1.56926
H	4.61884	-1.90081	-0.075
H	5.88303	-2.66342	0.91248
H	-0.67226	-0.05617	5.01328
H	-1.28353	2.27149	-3.36795
H	-5.32873	3.97322	-0.1798
H	4.01262	3.81111	-2.05629
H	2.69647	-5.19153	-1.51152
H	1.82775	0.05901	4.49821
H	-6.4298	-0.18471	1.92078

Supplementary Table 8. Coordinates of DFT optimized quintet structure.

Fe	0	0	0
C	-3.26232	0.7865	0.62659
C	-0.66916	-3.29468	0.60239
C	3.17478	-0.81606	-0.93997
C	0.75602	3.31657	-0.2434
N	-1.63644	-1.04533	0.49308
C	-2.90232	-0.55707	0.68918
C	-3.82495	-1.63125	1.0263
C	-3.08649	-2.78214	1.04544
C	-1.72439	-2.3979	0.70738
C	-3.52872	-4.18156	1.33527
C	-5.28573	-1.44542	1.2887
N	1.06156	-1.72655	-0.13012
C	0.63319	-2.97998	0.21385
C	1.69871	-3.9493	0.07483
C	2.79141	-3.25193	-0.38296
C	2.36813	-1.86788	-0.50787

C	1.59418	-5.40735	0.39115
C	4.16298	-3.77341	-0.65677
N	1.62448	1.04251	-0.52265
C	2.83979	0.53531	-0.91298
C	3.7862	1.60069	-1.17384
C	3.1324	2.77152	-0.88629
C	1.77647	2.40479	-0.50976
C	5.2095	1.42382	-1.59491
C	3.71922	4.14116	-0.84599
N	-1.06	1.72139	0.12602
C	-0.57068	2.996	0.02653
C	-1.62962	3.97142	0.21807
C	-2.77731	3.2525	0.43497
C	-2.39585	1.85199	0.3865
C	-1.44174	5.45525	0.16901
C	-4.16675	3.74967	0.68213
H	-4.30689	1.0291	0.81302
H	-0.88178	-4.33925	0.82203
H	4.17953	-1.05985	-1.27607
H	1.00728	4.37455	-0.27939
H	-3.37413	-4.84436	0.47083
H	-4.59451	-4.21973	1.59065
H	-2.97253	-4.61784	2.17831
H	-5.80469	-1.02963	0.41245
H	-5.77253	-2.39602	1.53777
H	2.50813	-5.94162	0.10744
H	0.7502	-5.87785	-0.1343
H	1.44402	-5.57794	1.46732
H	4.74369	-3.08239	-1.27809
H	5.6183	2.35467	-2.00712
H	5.31359	0.64597	-2.36406
H	5.85272	1.14258	-0.74792
H	2.95217	4.9232	-0.88628
H	-2.38579	5.98508	0.34401
H	-1.05687	5.78503	-0.80736
H	-0.72597	5.80054	0.92987
H	-4.54827	3.42587	1.66202
H	-4.21097	4.84525	0.65997
C	-0.02319	1.11657	-3.35371
S	-0.81587	-0.14824	-2.32286
C	-0.18562	-1.63446	-3.15128
H	-0.39275	1.05824	-4.38437
H	1.06751	1.01397	-3.32988
H	-0.57664	-2.4961	-2.60081

H	0.90932	-1.66233	-3.11948
H	-0.54158	-1.67407	-4.18755
N	0.5324	0.17183	2.00056
C	-0.28868	0.41042	3.00577
C	1.79104	0.07323	2.55182
N	0.39009	0.4714	4.18006
C	1.72334	0.25476	3.90889
S	4.80302	4.44234	0.62617
C	3.65418	4.01147	1.97686
C	4.40382	3.81889	3.30297
S	5.15703	-4.22244	0.83741
C	5.13457	-2.68207	1.8129
C	6.02909	-1.54842	1.25441
H	-1.35905	0.54501	2.91677
H	2.65426	-0.1215	1.92902
H	2.89642	4.7996	2.07853
H	3.14072	3.08168	1.70398
H	3.68799	3.56364	4.09562
H	4.93445	4.73108	3.60601
H	5.13816	3.00738	3.22445
H	4.09192	-2.3533	1.91624
H	5.48153	-2.9884	2.80827
H	5.98567	-0.66999	1.91407
H	5.69623	-1.23555	0.25977
H	7.0717	-1.88092	1.18125
H	-0.01622	0.64104	5.08961
H	-0.29502	2.08714	-2.92653
H	-4.87081	3.37691	-0.07621
H	4.39588	4.31284	-1.69393
H	4.12053	-4.72478	-1.20467
H	2.47745	0.247	4.68401
H	-5.46545	-0.75505	2.1264

Supplementary Table 9. Coordinates of DFT triplet structure at crossing point.

Fe	0	0	0
C	-0.98554	-3.27995	-0.15908
C	3.2766	-0.98752	0.13804
C	0.98843	3.27534	-0.15636
C	-3.2681	0.97921	0.23236
N	0.96829	-1.80093	-0.02932
C	0.39342	-3.03951	-0.12115
C	1.41845	-4.07246	-0.11929

C	2.62349	-3.42468	-0.00903
C	2.32111	-2.00253	0.04356
C	4.00224	-4.00402	0.04172
C	1.14679	-5.54023	-0.2208
N	1.80005	0.97166	0.00613
C	3.03582	0.39192	0.12469
C	4.07055	1.40729	0.18495
C	3.4285	2.61892	0.08296
C	2.0073	2.32425	-0.03403
C	5.53426	1.14492	0.34336
C	4.03351	3.98259	0.13153
N	-0.96817	1.79709	-0.00341
C	-0.39058	3.03634	-0.10808
C	-1.39943	4.0722	-0.02715
C	-2.59828	3.42732	0.17703
C	-2.31087	2.00201	0.16117
C	-1.14896	5.5453	-0.07839
C	-3.91432	4.05796	0.48025
N	-1.7948	-0.96977	-0.00452
C	-3.031	-0.39348	0.1305
C	-4.06826	-1.41558	0.12688
C	-3.42502	-2.6189	-0.01508
C	-2.00142	-2.32076	-0.08202
C	-5.5339	-1.14047	0.24996
C	-4.01041	-3.99373	-0.08784
H	-1.29932	-4.3202	-0.22354
H	4.31665	-1.30004	0.20972
H	1.29086	4.3162	-0.24022
H	-4.30651	1.28193	0.34956
H	4.61639	-3.66817	-0.80708
H	3.97761	-5.10012	0.01408
H	4.53388	-3.71028	0.95904
H	0.63731	-5.7934	-1.1622
H	2.07451	-6.12401	-0.18298
H	6.11485	2.07096	0.26179
H	5.90627	0.44388	-0.41829
H	5.76283	0.70859	1.32691
H	3.35063	4.74415	-0.2616
H	-2.07274	6.0967	-0.29357
H	-0.41718	5.80774	-0.85493
H	-0.76814	5.92711	0.88042
H	-4.75246	3.37229	0.31004
H	-6.11971	-2.06715	0.22275
H	-5.89785	-0.49835	-0.5659

H	-5.77427	-0.62796	1.19348
H	-3.65294	-4.63367	0.73276
H	-5.10526	-3.96784	-0.0292
C	-1.33552	0.91629	-3.11376
S	-0.06377	-0.18514	-2.43709
C	1.40497	0.56279	-3.19607
H	-1.37279	0.82794	-4.20587
H	-1.15137	1.95467	-2.81666
H	2.27124	-0.00323	-2.83903
H	1.51369	1.60819	-2.88672
H	1.34744	0.48822	-4.28841
N	0	0	2.06165
C	-0.23352	-1.04174	2.83807
C	0.22912	1.06474	2.90545
N	-0.16304	-0.68998	4.14712
C	0.13182	0.65442	4.20945
S	-4.03387	4.73374	2.20129
C	-3.5959	3.25123	3.1708
C	-3.26165	3.61757	4.62397
S	4.64517	4.52178	1.79199
C	3.1681	4.32992	2.84419
C	2.07386	5.40352	2.62679
H	-0.45353	-2.04232	2.48878
H	0.44637	2.04831	2.51032
H	-4.42761	2.53499	3.14052
H	-2.72597	2.77962	2.69804
H	-3.00547	2.70745	5.18252
H	-4.11122	4.09685	5.12793
H	-2.40698	4.30422	4.669
H	2.76871	3.31747	2.69817
H	3.55837	4.38345	3.8688
H	1.23547	5.2375	3.31834
H	1.67814	5.36401	1.60707
H	2.47914	6.40758	2.80163
H	-0.30073	-1.31013	4.93274
H	-2.29189	0.59243	-2.69085
H	-3.74326	-4.49787	-1.02823
H	-4.09134	4.94166	-0.14765
H	4.94333	4.03282	-0.48234
H	0.24672	1.17882	5.14807
H	0.50308	-5.89394	0.59837

Supplementary Table 10. Coordinates of DFT quintet structure at crossing point.

Supplementary Note 6: Temporal alignment of the Kβ XES datasets from different experiments

The femtosecond XES datasets of the two different experiments were temporally aligned as shown in Supplementary Fig. 10. The same temporal shift was then used to align the XSS data from experiment #2 with the XES data from experiment #1.

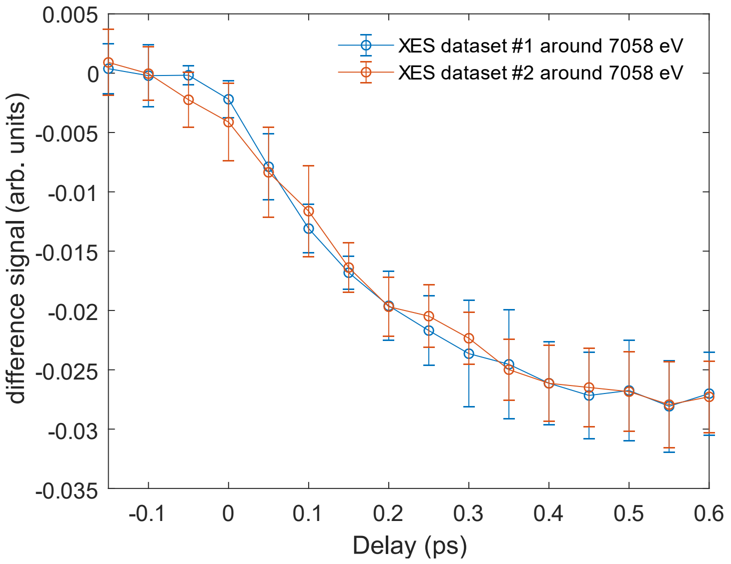

Supplementary Fig. 10. Scaled kinetic traces of the two Kβ XES datasets integrated in a narrow pixel range (7 pixels) around 7058 eV after temporal alignment of the two experiments. Error bars reflect the standard deviation of the individual pixel signals at each time delay.

Supplementary References

1. Mara MW*, et al.* Metalloprotein entatic control of ligand-metal bonds quantified by ultrafast x-ray spectroscopy. *Science* **356**, 1276 (2017).

2. Wang W*, et al.* Femtosecond Multicolor Pump−Probe Spectroscopy of Ferrous Cytochrome c. *The Journal of Physical Chemistry B* **104**, 10789-10801 (2000).

3. Bräm O, Consani C, Cannizzo A, Chergui M. Femtosecond UV Studies of the Electronic Relaxation Processes in Cytochrome c. *The Journal of Physical Chemistry B* **115**, 13723-13730 (2011).

4. Burnham K, Anderson D. Model Selection and Multimodel Inference. *A Practical Information-theoretic Approach*, (2004).

5. Lewis F, Butler A, Gilbert L. A unified approach to model selection using the likelihood ratio test. *Methods in Ecology and Evolution* **2**, 155-162 (2011).

6. Lemke HT*, et al.* Coherent structural trapping through wave packet dispersion during photoinduced spin state switching. *Nature Communications* **8**, 15342 (2017).

7. Kjaer KS*, et al.* Introducing a standard method for experimental determination of the solvent response in laser pump, X-ray probe time-resolved wide-angle X-ray scattering experiments on systems in solution. *Physical Chemistry Chemical Physics* **15**, 15003-15016 (2013).

8. Cammarata M*, et al.* Tracking the structural dynamics of proteins in solution using time-resolved wide-angle X-ray scattering. *Nat Meth* **5**, 881-886 (2008).

9. Banci L, Bertini I, Huber JG, Spyroulias GA, Turano P. Solution structure of reduced horse heart cytochrome c. *Journal of biological inorganic chemistry : JBIC : a publication of the Society of Biological Inorganic Chemistry* **4**, 21-31 (1999).

10. Haldrup K, Christensen M, Meedom Nielsen M. Analysis of time-resolved X-ray scattering data from solution-state systems. *Acta Crystallographica Section A* **66**, 261-269 (2010).

11. Brown PJ, Fox AG, Maslen EN, O'Keefe MA, Willis BTM. Intensity of diffracted intensities. In: *International Tables for Crystallography Volume C: Mathematical, physical and chemical tables* (ed Prince E). Springer Netherlands (2004).

12. Zhang W*, et al.* Tracking excited-state charge and spin dynamics in iron coordination complexes. *Nature* **509**, 345-348 (2014).
